# NADPH- and NADH-dependent metabolism of and DNA adduct formation by benzo[*a*]pyrene catalyzed with rat hepatic microsomes and cytochrome P450 1A1

**DOI:** 10.1007/s00706-016-1713-y

**Published:** 2016-03-09

**Authors:** Marie Stiborová, Radek Indra, Michaela Moserová, Miroslav Šulc, Petr Hodek, Eva Frei, Heinz H. Schmeiser, Volker M. Arlt

**Affiliations:** Department of Biochemistry, Faculty of Science, Charles University, Albertov 2030, 128 40 Prague 2, Czech Republic; Division of Radiopharmaceutical Chemistry, German Cancer Research Center (DKFZ), Im Neuenheimer Feld 280, 69120 Heidelberg, Germany; Analytical and Environmental Sciences Division, MRC-PHE Centre for Environment and Health, King’s College London, London, SE1 9NH UK

**Keywords:** DNA, Enzymes, Coenzymes, High-pressure liquid chromatography

## Abstract

**Abstract:**

Benzo[*a*]pyrene (BaP) is a human carcinogen that covalently binds to DNA after metabolic activation by cytochrome P450 (CYP) enzymes. Here we investigated the efficiencies of rat hepatic microsomes and rat recombinant CYP1A1 expressed with its reductase, NADPH:CYP oxidoreductase (POR), NADH:cytochrome *b*_*5*_ reductase, epoxide hydrolase and/or cytochrome *b*_*5*_ in Supersomes™ to metabolize this carcinogen. We also studied the effectiveness of coenzymes of two of the microsomal reductases, NADPH as a coenzyme of POR, and NADH as a coenzyme of NADH:cytochrome *b*_*5*_ reductase, to mediate BaP metabolism in these systems. Up to eight BaP metabolites and two DNA adducts were generated by the systems, both in the presence of NADPH and NADH. Among BaP metabolites, BaP-9,10-dihydrodiol, BaP-4,5-dihydrodiol, BaP-7,8-dihydrodiol, BaP-1,6-dione, BaP-3,6-dione, BaP-9-ol, BaP-3-ol, and a metabolite of unknown structure were formed by hepatic microsomes and rat CYP1A1. One of two DNA adducts formed by examined enzymatic systems (rat hepatic microsomes and rat CYP1A1) was characterized to be 10-(deoxyguanosin-*N*^2^-yl)-7,8,9-trihydroxy-7,8,9,10-tetrahydrobenzo[*a*]pyrene (dG-*N*^2^-BPDE), while another adduct has similar chromatographic properties on polyethylaneimine–cellulose thin layer chromatography to a guanine adduct derived from reaction with 9-hydroxy-BaP-4,5-oxide. In the presence of either of the reductase cofactors tested, NADPH or NADH, cytochrome *b*_*5*_ stimulated CYP1A1-mediated formation of both BaP-DNA adducts. The results demonstrate that NADH can act as a sole electron donor for both the first and the second reduction of CYP1A1 during its reaction cycle catalyzing oxidation of BaP, and suggest that the NADH:cytochrome *b*_*5*_ reductase as the NADH-dependent reductase might substitute POR in this enzymatic system.

**Graphical abstract:**

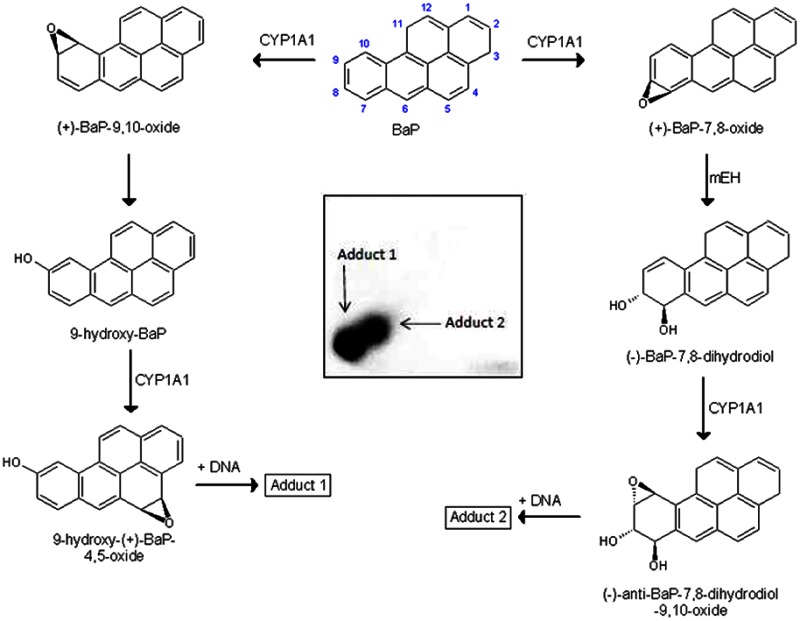

## Introduction

**Fig. 1:**
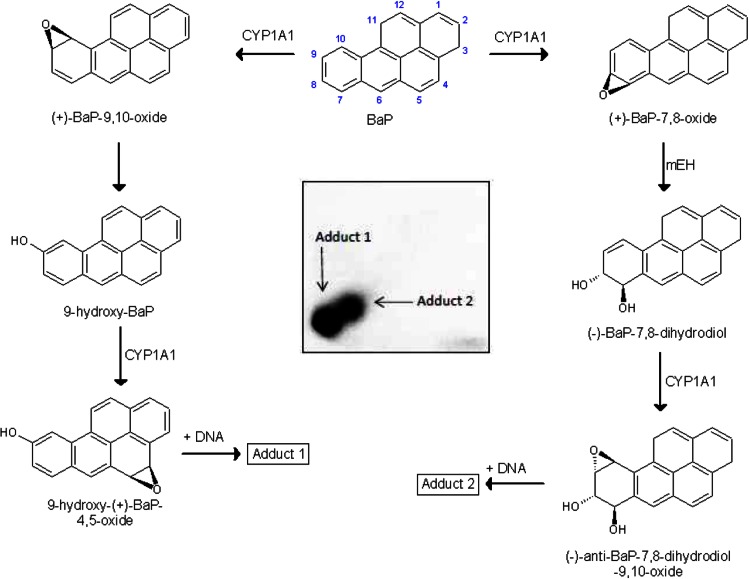
Proposed pathways of biotransformation and DNA adduct formation of BaP catalyzed by CYP1A1 and *m*EH. As shown in the *left* part of the figure, the two-step activation process by CYP1A1 leads to the formation of 9-hydroxy-BaP-4,5-oxide that can react with deoxyguanosine in DNA (adduct 1). As shown in the *right* part of the figure, the typical three-step activation process by CYP1A1 followed by hydrolysis by *m*EH leads to BPDE which forms dG-*N* ^2^-BPDE (adduct 2) (adopted from [15]). *Inset* autoradiographic profile of BaP-DNA adducts formed by hepatic microsomes of rats pretreated with BaP as evaluated by thin-layer chromatography ^32^P-postlabeling as described previously [15]; the *arrows* show adducts 1 and 2

Benzo[*a*]pyrene (BaP, Fig. 1) is a polycyclic aromatic hydrocarbon (PAH) that has been classified as human carcinogen (group 1) by the International Agency for Research on Cancer [1]. BaP requires metabolic activation catalyzed by cytochrome P450 (CYP) enzymes prior to reaction with DNA [2]. Among CYP enzymes, CYP1A1 is one of the most important CYPs involved in the metabolic activation of BaP to species forming DNA adducts [2, 3], in combination with microsomal epoxide hydrolase (*m*EH). First, CYP1A1 oxidizes BaP to an epoxide (oxide) that is then converted to a dihydrodiol by *m*EH (i.e. BaP-7,8-dihydrodiol); then further bio-activation by CYP1A1 leads to the ultimately reactive species, BaP-7,8-dihydrodiol-9,10-oxide (BPDE) that can react with DNA, forming adducts preferentially at guanine residues (Fig. 1). The 10-(deoxyguanosin-*N*^2^-yl)-7,8,9-trihydroxy-7,8,9,10-tetrahydrobenzo[*a*]pyrene (dG-*N*^2^-BPDE) adduct is the major product of the reaction of BPDE with DNA in vitro and in vivo [4–8].

BaP is, however, also oxidized to other metabolites such as other dihydrodiols, BaP-diones, and hydroxylated metabolites [2, 6, 9–12]. Even though most of these metabolites are detoxification products, BaP-9-ol is a precursor of 9-hydroxy-BaP-4,5-oxide that can form another adduct with deoxyguanosine in DNA (Fig. 1) [7, 8, 13–15].

The CYP enzymes, including CYP1A1, are a component of a mixed-function oxidase (MFO) system located in the membrane of endoplasmic reticulum. This enzymatic system contains beside the CYPs also another enzyme, NADPH:cytochrome P450 oxidoreductase (POR), and cytochrome *b*_*5*_ accompanied with its NADH:cytochrome *b*_*5*_ reductase. Via the activation of molecular oxygen, this multienzyme system catalyzes the monooxygenation of a variety of xenobiotics, including BaP [16]. The oxygen is activated in the active center of CYPs by two electrons transferred from NADPH and/or NADH by means of POR and/or cytochrome *b*_*5*_, respectively. It is generally accepted that NADPH serves as a donor of electrons for both reductions of CYP mediated by POR in the MFO reaction cycle [16]. The second electron may, however, also be provided by the system of NADH:cytochrome *b*_*5*_ reductase with cytochrome *b*_*5*_ and NADH, and cytochrome *b*_*5*_ seems to play also additional roles in the MFO system [16–23]. It should be noted that although POR is considered as an essential constituent of the electron transport chain towards CYP [16], its exact role in the CYP-mediated reaction cycle is still not clearly established. Recently we found that in two mouse models treated with BaP [i.e. Hepatic P450 Reductase Null (HRN) and Reductase Conditional Null (RCN)], in which the expression of POR has been permanently or conditionally deleted in hepatocytes leading to a lack of almost all POR activity, the levels of the CYP- and *m*EH-mediated dG-*N*^2^-BPDE adducts in livers were higher than in BaP-treated wild-type mice [7, 8, 15]. This finding indicates a contribution of either activation of BaP in cells other than hepatocytes in the liver (e.g. Kupffer or endothelial cells) [24], or by non CYP enzymes (such as prostaglandin H synthetase and lipoxygenases) [25, 26], or combinations of these mechanisms.

Moreover, this might also suggest that another reductase can contribute to the CYP-mediated BaP oxidation metabolism in these animal models. Nevertheless, the questions which of the cellular reductases might play this role and whether NADH:cytochrome *b*_*5*_ reductase as a component of the MFO system might be one of such reductases, remain to be answered. Therefore, to resolve whether NADH:cytochrome *b*_*5*_ reductase can substitute POR in the CYP-reaction cycle oxidizing BaP is a challenge for the future research. In this study, we investigated the role of NADH, a coenzyme of NADH:cytochrome *b*_*5*_ reductase in the CYP1A1-mediated metabolism of BaP and the formation of BaP-DNA adduct in vitro, and compared its efficiency with NADPH, a coenzyme of the POR-dependent system. Here, we also investigated the effect of cytochrome *b*_*5*_ on these processes. Rat hepatic microsomes and rat recombinant CYP1A1 expressed in Supersomes™ were used as models for such a study.

## Results and discussion

### Oxidation of BaP by rat hepatic microsomes and rat CYP1A1 expressed in Supersomes™ in the presence of NADPH and NADH

The metabolism of PAHs has been intensively studied over the past decades [2] and various studies have examined the role of the CYP systems, particularly CYP1A1, of several species to metabolize BaP [2, 6–8, 15, 23, 27]. However, because the exact participation of the POR/NADPH system as a donor of electrons for the reduction of CYP1A1 in its reaction cycle oxidizing BaP is still not fully resolved [7, 8, 15], here we investigated this feature. Rat CYP systems, shown to mimic, to some extent, the BaP metabolism by human CYPs [7, 15, 23], were used in our experiments. Two enzymatic systems were utilized to investigate their efficiencies to metabolize BaP: (1) microsomes isolated from livers of control (untreated) rats and rats pretreated with inducers of several CYP enzymes and (2) microsomes of baculovirus-infected insect cells (Supersomes™) containing rat recombinant CYP1A1 expressed with POR, NADH:cytochrome *b*_*5*_ reductase, *m*EH and/or cytochrome *b*_*5*_. In the CYP1A1 experimental system used, either basic CYP1A1-Supersomes™ or those reconstituted with purified cytochrome *b*_*5*_ were used. NADPH and NADH, cofactors of two microsomal reductases POR and NADH:cytochrome *b*_*5*_ reductase, respectively, were utilized as electron donors for CYP-mediated BaP oxidation. The BaP metabolite profile formed by individual hepatic microsomes and rat CYP1A1 in Supersomes™ was determined by HPLC analysis and BaP metabolites were identified by NMR and/or mass spectrometry as described previously [15].

**Fig. 2:**
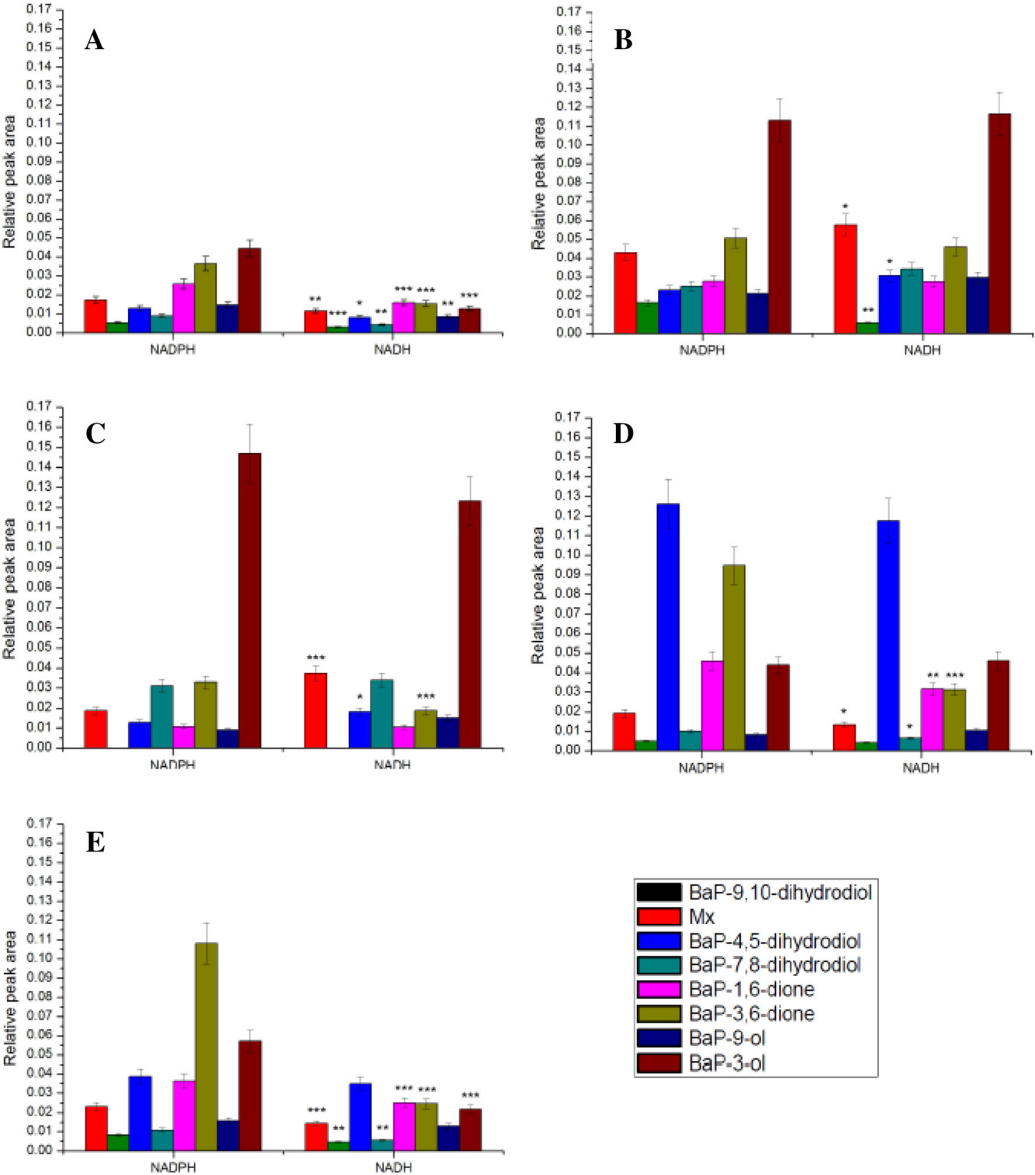
Metabolism of BaP in microsomes isolated from livers of control rats (**a**) or rats pretreated with Sudan I (**b**), BaP (**c**), PB (**d**) and PCN (**e**) in the presence of either NADPH or NADH. Values represent mean ± SD from three parallel measurements. *P < 0.05, **P < 0.01, ***P < 0.001 (Student’s *t* test), significantly different from incubations in the presence of NADPH

Up to eight BaP metabolites were formed in hepatic microsomes of untreated- or pretreated-rats, both in the presence of NADPH and NADH. They were structurally characterized previously [15] to be: BaP-9,10-dihydrodiol (M1), BaP-4,5-dihydrodiol (M2), BaP-7,8-dihydrodiol (M3), BaP-1,6-dione (M4), BaP-3,6-dione (M5), BaP-9-ol (M6), and BaP-3-ol (M7). In addition to these BaP metabolites, a metabolite of unknown structure (Mx) was also formed. Essentially no BaP metabolites were found when NADPH or NADH was omitted from the incubation mixtures containing the tested microsomes (data not shown). The amounts of BaP metabolites formed and their profile obtained were dependent on the individual microsomes and on the presence of NADPH or NADH (Fig. 2).

In the presence of NADH, the amounts of BaP metabolites formed in incubations of BaP with hepatic microsomes isolated from untreated rats (Fig. 2a) and rats pretreated with pregnenolone-16α-carbonitrile (PCN) (Fig. 2e), an inducer of rat CYP3A, were significantly lower than in the presence of NADPH; the amounts were up to five times lower, depending on the individual metabolites. Also the patterns of individual BaP metabolites formed by these microsomes in the presence of either coenzyme differed significantly (Fig. 2). The microsomes of rats pretreated with inducers of CYP1A enzymes, Sudan I [28], BaP [7, 8], metabolized BaP in the presence of NADH with essentially the same efficiency as in that of NADPH (Fig. 2b, c). In the presence of NADH, some of BaP metabolites such as BaP-9,10-dihydrodiol, BaP-4,5-dihydrodiol, BaP-7,8-dihydrodiol, and BaP-diones were generated even at higher levels than in the presence of NADPH. In contrast, treatment of rats with Sudan I and BaP led to a decrease in the formation of a metabolite with the unknown structure (Mx) (Fig. 2b, c). Using microsomes of rats where CYPs of the 2B subfamily were induced by phenobarbital (PB) the amounts of BaP metabolites formed in the presence NADH were also similar to those formed in the presence of NADPH. But the formation of BaP-9,10-dihydrodiol, BaP-7,8-dihydrodiol, BaP-1,6-dione, and BaP-3,6-dione by these microsomes was significantly lower than in the presence of NADPH (Fig. 2d). Induction of CYP2B also resulted in the formation of significantly higher amounts of BaP-4,5-dihydrodiol which is formed in other test microsomes as a minor BaP metabolite. This indicates a role of CYP2B in the formation of this BaP metabolite in rat liver microsomes.

The results of these experiments demonstrate that NADH acts a donor of electrons for the reduction of the CYPs during BaP oxidation in rat liver microsomes, predominantly in those from liver of rats pretreated with inducers of CYP1A and 2B enzymes, at essentially the same efficiency as NADPH. They also suggest that this cofactor can serve even as an exclusive donor of electrons for CYP in its catalytic cycle, functioning independently of NADPH and POR. This suggestion is strongly supported by the finding that NADH does not function as a coenzyme of POR when cytochrome *c* is used as a substrate (data not shown).

**Fig. 3:**
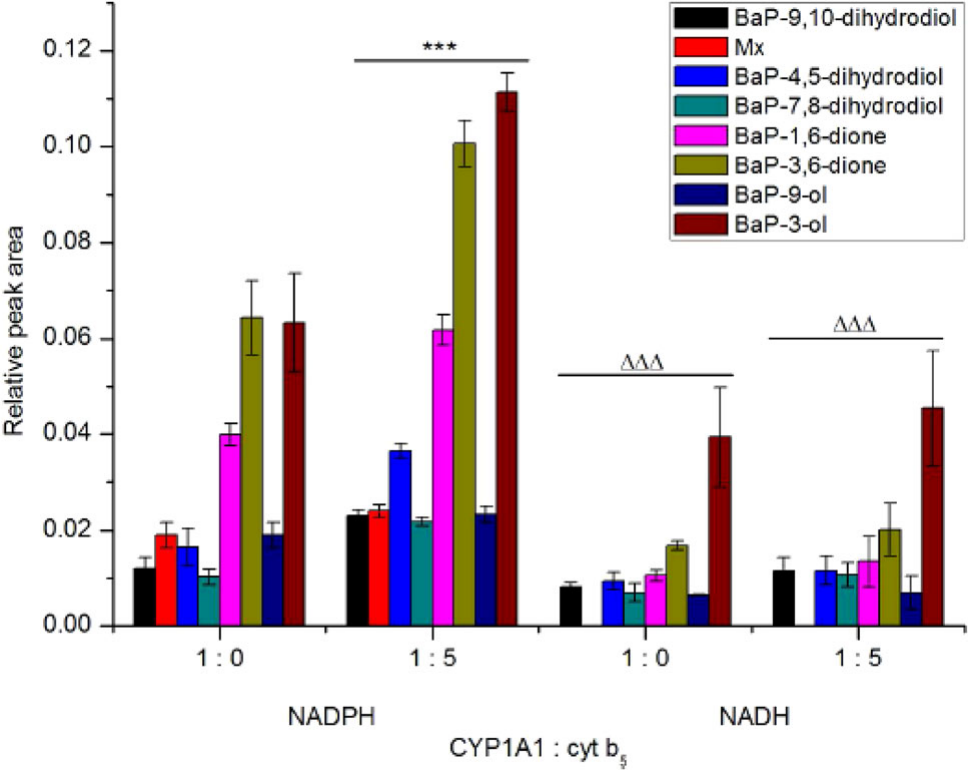
Metabolism of BaP by rat recombinant CYP1A1 in Supersomes™ in the presence of either NADPH or NADH and the effect of cytochrome *b*_*5*_ on this metabolism. Beside CYP1A1 and POR over-expressed in Supersomes™, this enzymatic system also contained *m*EH. Values represent mean ± SD from three parallel measurements. ***P < 0.001 (Student’s *t* test), significantly different from incubations without cytochrome *b*_*5*_; ^ΔΔΔ^Δ*P* < 0.001 (Student’s *t* test), significantly different from incubations in the presence of NADPH

Rat recombinant CYP1A1 expressed in the Supersomal enzymatic system oxidized BaP to the same metabolites as rat hepatic microsomes, again both in the presence of NADPH and NADH. However, in this system NADH exhibited lower effectiveness than NADPH to act as a donor of electrons for the reduction of CYP1A1. Addition of cytochrome *b*_*5*_ to the incubation mixtures led to an increase in BaP oxidation by NADPH-dependent CYP1A1-mediated metabolism of this carcinogen, while its stimulation effect on BaP oxidation in the presence of NADH was not statistically significant (Fig. 3).

### Formation of BaP-DNA adducts by rat hepatic microsomes and rat CYP1A1 expressed in Supersomes™ in the presence of NADPH and NADH

In further experiments, DNA adduct formation by BaP incubated with rat hepatic microsomes and rat CYP1A1 were analyzed. We utilized microsomes isolated from the livers of untreated and Sudan I- and BaP-pretreated rats, of which the latter two are the most effective in catalyzing BaP metabolism (see Fig. 2), as well as rat recombinant CYP1A1 expressed with other enzyme components of the MFO system of Supersomes™ were utilized.

**Fig. 4:**
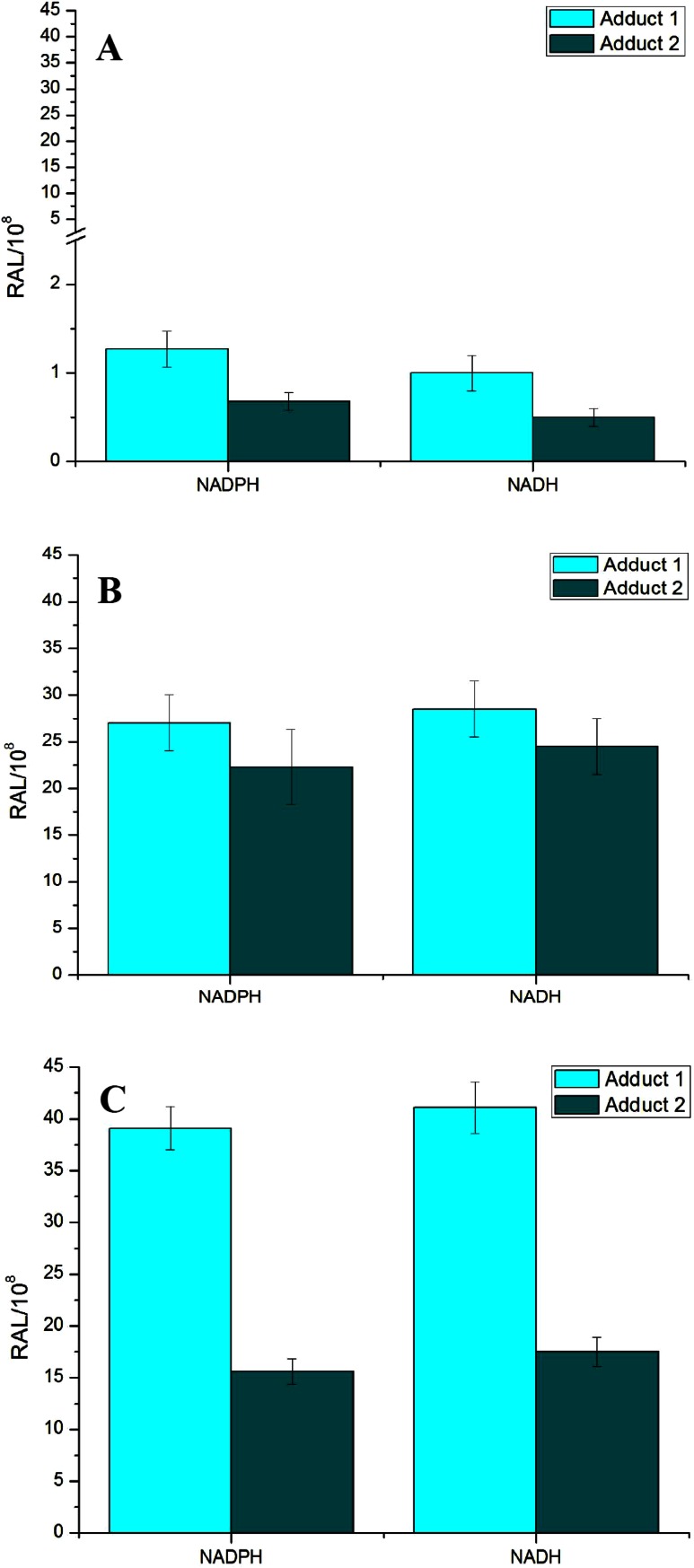
DNA adduct formation by BaP activated with microsomes isolated from livers of control rats (**a**) or rats pretreated with Sudan I (**b**) or BaP (**c**) in the presence of either NADPH or NADH. Comparison with previous ^32^P-postlabeling studies [15, 29] showed that adduct spot 1 has similar chromatographic properties on PEI-cellulose TLC to a guanine adduct derived from reaction with 9-hydroxy-BaP-4,5-oxide [15]. Adduct 2 is dG-*N* ^2^-BPDE adduct. For all panels, values represent mean total relative adduct labeling (RAL) ± SD (*n* = 3; analyses of three independent in vitro incubations)

**Fig. 5:**
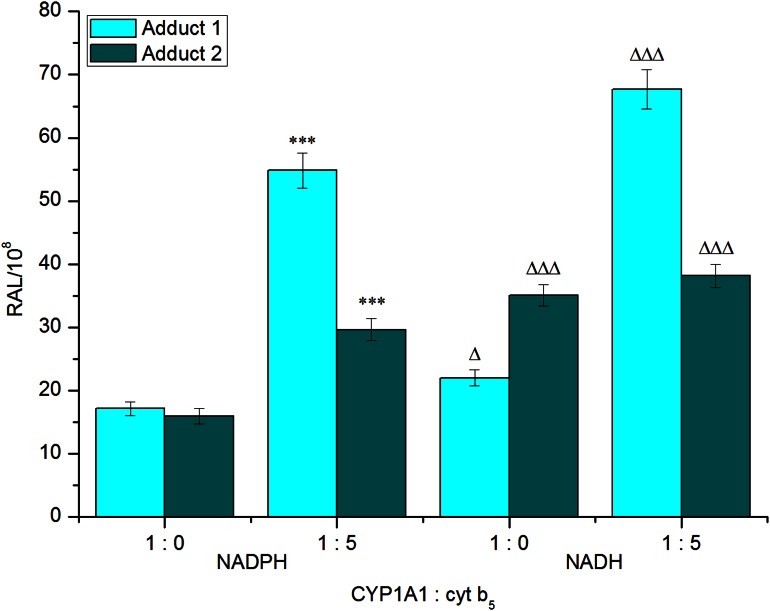
DNA adduct formation by BaP activated with rat recombinant CYP1A1 in Supersomes™ in the presence of either NADPH or NADH and the effect of cytochrome *b*_*5*_ on this reaction. Beside CYP1A1 and POR over-expressed in Supersomes™, this enzymatic system also contained *m*EH. Values represent mean total relative adduct labeling (RAL) ± SD (*n* = 3; analyses of three independent in vitro incubations). ***P < 0.001 (Student’s *t* test), levels of BaP-adducts 1 and 2 significantly different from incubations without cytochrome *b*_*5*_; ^ΔΔΔ^Δ*P* < 0.001 (Student’s *t* test), levels of BaP-adducts 1 and 2 significantly different from incubations in the presence of NADPH

Two DNA adducts determined by the ^32^P-postlabeling method [7, 8, 15] (see adduct spots 1 and 2 in insert of Fig. 1) were formed by BaP activated with the test microsomes and CYP1A1 in Supersomes™, again in the presence of both NADPH and NADH (Figs. 4, 5). Comparison with previous ^32^P-postlabeling studies [15, 29] showed that adduct spot 1 has similar chromatographic properties on polyethylenimine-cellulose thin-layer chromatography to a guanine adduct derived from reaction with 9-hydroxy-BaP-4,5-epoxide (see adduct spot 1 in insert of Fig. 1), The other adduct was formed from BaP-7,8-dihydrodiol-9,10-oxide with guanine in DNA and characterized as the dG-*N*^2^-BPDE adduct (adduct spot 2 in insert of Fig. 1) [15, 29]. Whereas the levels of these BaP-DNA adducts were dependent on the type of microsomes examined, being highest in microsomes of rats treated with BaP, essentially no differences were seen in their dependence on the presence of cofactors used, NADPH or NADH (Fig. 4). Pretreatment of rats with inducers of CYP1A1, Sudan I [28], and BaP [7, 8], which is the predominant enzyme metabolizing BaP [2, 6], resulted in up to more than fortyfold higher levels of BaP-DNA adducts in microsomal incubations (Fig. 4). The increase in potency of microsomes, where CYP1A1 was induced, to form BaP-DNA adducts corresponded to higher efficiencies of these microsomes to metabolize BaP (compare Figs. 2, 4).

Rat recombinant CYP1A1 expressed in Supersomes™ was also active to form BaP-DNA adducts. The adducts formed were the same as those generated by hepatic microsomes, again both in the presence of NADPH and NADH (Fig. 5). Interestingly, in these experiments NADH was even more efficient than NADPH to serve as a donor of electrons for the reduction of CYP1A1 in its catalytic cycle. However, it should be noted that when the oxidation of BaP to its metabolite products was analyzed in this CYP1A1 system (Supersomes™), NADH was less efficient than NADPH (see Fig. 3). Now we can only speculate on the reasons that can cause this phenomenon. One of them might be the effects of different experimental conditions used for the incubations utilized for analysis of BaP metabolites and BaP-DNA adducts (i.e. incubation times, concentrations of CYP1A1 and BaP, see the “Experimental” section).

Similar to an increase in the oxidation of BaP by CYP1A1-Supersomes™ by cytochrome *b*_*5*_, addition of the protein to this system produced also elevated levels of BaP-DNA adducts generated by rat CYP1A1. Here, increases in the formation of BaP-DNA adducts by cytochrome *b*_*5*_ were not dependent on the type of reduced cofactors (i.e. NADPH or NADH) as elevated levels of BaP-DNA adducts were mediated by both cofactors (Fig. 5).

## Conclusion

The results of this study demonstrate that rat CYP1A1 present in the natural microsomal system of rat liver or in microsomes of baculovirus-infected insect cells (Supersomes™), where also other components of the MFO-system such as reductases POR and NADH:cytochrome *b*_*5*_ reductase, cytochrome *b*_*5*_ and *m*EH are expressed, metabolize the carcinogenic environmental pollutant BaP. It should be, moreover, emphasized that this metabolism occurs not only in the presence of the cofactor of POR, NADPH, but also in the presence of NADH, which is an exclusive coenzyme of the other microsomal reductase, NADH:cytochrome *b*_*5*_ reductase. Therefore, the reaction proceeds without any participation of NADPH, and hence without POR. These results bring a novel view on the mechanism of the catalytic cycle of rat CYP1A1 during oxidation of BaP. The data indicate that the catalytic cycle of the CYP reactions in the MFO system can proceed by different ways than it was suggested for the generally accepted mechanism. Namely, it was supposed that in the catalytic cycle of the microsomal CYP system electrons are transferred from NADPH via POR, while cytochrome *b*_*5*_ can contribute reducing power to this system after being reduced by NADH cytochrome *b*_*5*_:reductase in the presence of NADH. Whereas the first reduction of this enzyme was supposed to be mediated exclusively by the NADPH/POR system, the second one might be also catalyzed by the system of NADH/NADH:cytochrome *b*_*5*_ reductase [16, 30–33]. Here, we show that even in the absence of NADPH and its substitution by NADH, rat CYPs oxidize BaP in test microsomes. This reaction was found both in the natural rat hepatic microsomes and in microsomes of insect cells containing rat recombinant CYP1A1 (Supersomes™). Such NADH-mediated activity of these CYP systems was proved by the formation of BaP metabolic products as well as by generation of BaP-DNA adducts which were the same as in the system where NADPH was used as the donor of electrons. However, even though the role of the NADH/NADH:cytochrome *b*_*5*_ reductase system in both two reductions of rat CYP1A1 was proved in our study, this finding needs to be explored by further investigations. Furthermore, whether the observed phenomenon is valid not only for rat CYP1A1 but also for its human ortholog, is waiting for future research. For such studies, the enzymatic system containing pure rat and human CYP1A1 reconstituted with NADH:cytochrome *b*_*5*_ reductase and cytochrome *b*_*5*_ in liposomes [20, 34–36] is planned to be utilized. Preparation of these pure enzymes is therefore under way in our laboratory [37, 38; Milichovský et al., unpublished results].

## Experimental

BaP (CAS no. 50-32-8; purity ≥96 %), NADH and NADPH disodium and tetrasodium salts of ~95 and ~98 % purity, respectively, were obtained from Sigma Chemical Co (St Louis, MO, USA).

### Isolation of cytochrome b_5_

Cytochrome *b*_*5*_ was isolated from rabbit liver microsomes to more than 90 % purity as estimated the sodium dodecyl sulfate–polyacrylamide gel electrophoresis as described by Ross [36]. The purified cytochrome *b*_*5*_ was further used for the reconstitution experiments.

### Animal experiments and isolation of microsomes

All animal experiments were conducted in accordance with the Regulations for the Care and Use of Laboratory Animals (311/1997, Ministry of Agriculture, Czech Republic), which is in compliance with the Declaration of Helsinki. Male Wistar rats (~125–150 g, AnLab, Czech Republic) placed in cages in temperature- and humidity-controlled rooms were acclimatized for 5 days and maintained at 22 °C with a 12 h light/dark period. Standardized diet (ST-1 diet from Velaz, Czech Republic) and water were provided ad libitum. Rats were treated with inducers of CYP1A (Sudan I, BaP), CYP2B (PB), and CYP3A (PCN) as follows: (1) ten 5-week-old male Wistar rats (~125–150 g) were injected i.p. with 20 mg/kg b.w. Sudan I in maize oil once a day for three consecutive days as reported previously [28]. Animals in the control group received the same volume of maize oil on 3 days. Rats were killed 24 h after the last treatment by cervical dislocation; (2) ten 5-week-old male Wistar rats (~125–150 g) were treated p.o. by gastric gavages with a single dose of 150 mg/kg b.w. BaP dissolved in 1 cm^3^ sunflower oil as shown previously [39]. Animals in the control group were treated with 1 cm^3^ of sunflower oil only. Rats were killed 24 h after the last treatment by cervical dislocation; (3) ten 5-week-old male Wistar rats (~125–150 g) pretreated with PB (0.1 % in drinking water for 6 days) as described previously [40]. Animals in the control group received drinking water. Rats were killed after treatment by cervical dislocation; (4) ten 5-week-old male Wistar rats (~125–150 g) were injected i.p. with 50 mg/kg b.w. PCN dissolved in maize oil for four consecutive days as reported previously [41, 42]. Animals in the control group received the same volume of maize oil. Rats were killed 24 h after the last treatment by cervical dislocation. For all treatment groups, livers of the animals were removed immediately after killing, frozen in liquid nitrogen, and stored at −80 °C until isolation of microsomal fractions. Pooled microsomes were prepared from ten rat livers/group as reported [28, 40, 43] and used for experiments of our present study. As the control microsomes, those from rats treated with 1 cm^3^ of sunflower oil (by gavage, see above) were utilized. Activities of CYP marker substrates in these control microsomes did not differ significantly from those in other control microsomes. Microsomal fractions were stored at −80 °C until analysis. Protein concentrations in the microsomal fractions were assessed using the bicinchoninic acid protein assay with bovine serum albumin as a standard [44].

### Incubations to study metabolism of BaP in microsomes or rat recombinant CYP1A1 in Supersomes™

Incubation mixtures used for studying BaP metabolism by rat hepatic microsomes contained 100 mmol dm^−3^ potassium phosphate buffer (pH 7.4), 1 mmol dm^−3^ NADPH or NADH and 0.05 mmol dm^−3^ BaP (dissolved in 0.005 cm^3^ DMSO) and 0.5 mg of microsomal proteins of all prepared microsomes in a final volume of 0.5 cm^3^. The same amount of a solvent (DMSO) was used in control incubations without BaP. Under these DMSO concentrations, no inhibition of the NADPH-dependent CYP-catalyzed oxidation of several substrates has been found [7, 8, 15, 20, 23, 28]. The reaction was initiated by adding the NADPH or NADH. In alternate experiments 50 nmol dm^−3^ rat recombinant CYP1A1 present with its reductase, POR, in Supersomes™ (Gentest Corp., Woburn, MI, USA) was used instead of microsomal protein. Supersomes™ are microsomes isolated from insect cells that have been transfected with a baculovirus construct containing cDNA of human CYP1A1 and POR, which were therefore over-expressed in these microsomes. However, because they are microsomes (particles of broken endoplasmic reticulum), other enzymes (proteins) of the membrane of endoplasmic reticulum (NADH:cytochrome *b*_*5*_ reductase, microsomal EH (*m*EH) and cytochrome *b*_*5*_) are also expressed, at the basic levels, in these Supersomes™ (Gentest Corp., Woburn, MI, USA). In the experiments, where the effect of cytochrome *b*_*5*_ on enzymatic reaction catalyzed by human CYP1A1 in used Supersomes™, was investigated, a pure cytochrome *b*_*5*_ protein was added under the defined concentrations and reconstituted with Supersomes™ (see Figs. 3, 5). Enzyme reconstitution utilizing this CYP system in Supersomes™ and purified cytochrome *b*_*5*_ was performed as described elsewhere [15, 20, 21, 23, 34]. Control incubations were carried out either without microsomes (or without CYP1A1), or without cofactors or without BaP. After incubation (37 °C, 20 min), 0.005 cm^3^ phenacetin (1 mmol dm^−3^) in methanol was added as an internal standard. BaP metabolism by microsomes has been shown to be linear up to 30 min of incubation [15]. BaP metabolites were extracted twice with ethyl acetate (2 × 1 cm^3^), solvent evaporated to dryness, residues dissolved in 0.025 cm^3^ methanol and BaP metabolites separated by HPLC as reported [15, 45]. BaP metabolite peaks were collected and analyzed by HPLC by comparison with metabolic standards whose structure was determined by NMR and/or mass spectrometry as described recently [15].

### Determination of DNA adduct formation by BaP by ^32^P-postlabeling

Incubation mixtures used to assess DNA adduct formation by BaP [15] activated with all prepared rat hepatic microsomes or rat recombinant CYP1A1 consisted of 50 mmol dm^−3^ potassium phosphate buffer (pH 7.4), 1 mmol dm^−3^ NADPH or NADH, 0.5 mg of microsomal proteins (or 100 nmol dm^−3^ rat recombinant CYP1A1 in Supersomes™), 0.1 mmol dm^−3^ BaP (dissolved in 0.0075 cm^3^ DMSO), and 0.5 mg of calf thymus DNA in a final volume of 0.75 cm^3^. The reaction was initiated by adding 0.1 mmol dm^−3^ BaP. Incubations at 37 °C containing BaP were carried out for 60 min. BaP-DNA adduct formation has been shown to be linear up to 90 min [7, 15]. Control incubations were carried out either without microsomes (or CYP1A1), or without NADPH (or NADH), or without DNA, or without BaP. After the incubation, DNA was isolated from the residual water phase by the phenol/chloroform extraction method. DNA adducts were analyzed with the nuclease P1 version of the ^32^P-postlabeling technique [7, 8, 15]. Resolution of the adducts by thin-layer chromatography using polyethylenimine-cellulose plates (Macherey and Nagel, Düren, Germany) was carried out as described [15, 39]. DNA adduct levels (RAL, relative adduct labeling) were calculated as described [46].

### Statistical analyses

For statistical data analysis we used Student’s *t* test. All *P* values are two-tailed and considered significant at the 0.05 level.
